# A Survey of the Union of European Neonatal and Perinatal Societies on Neonatal Respiratory Care in Neonatal Intensive Care Units

**DOI:** 10.3390/children11020158

**Published:** 2024-01-26

**Authors:** Corrado Moretti, Camilla Gizzi, Luigi Gagliardi, Flavia Petrillo, Maria Luisa Ventura, Daniele Trevisanuto, Gianluca Lista, Raffaele L. Dellacà, Artur Beke, Giuseppe Buonocore, Antonia Charitou, Manuela Cucerea, Boris Filipović-Grčić, Nelly Georgieva Jeckova, Esin Koç, Joana Saldanha, Manuel Sanchez-Luna, Dalia Stoniene, Heili Varendi, Giulia Vertecchi, Fabio Mosca

**Affiliations:** 1 Department of Pediatrics, Policlinico Umberto I, Sapienza University, 00185 Rome, Italy; 2 Union of European Neonatal and Perinatal Societies (UENPS), 20143 Milan, Italy; camilla.gizzi@aslroma2.it (C.G.); gianluca.lista@asst-fbf-sacco.it (G.L.); beke.artur@med.semmelweis-univ.hu (A.B.); giuseppe.buonocore@unisi.it (G.B.); neogna@reamaternity.gr (A.C.); manuela.cucerea@umfst.ro (M.C.); boris.filipovic-grcic@kbc-zagreb.hr (B.F.-G.); njekova@medfac.mu-sofia.bg (N.G.J.); esinkoc@gazi.edu.tr (E.K.); joana.saldanha@hbeatrizangelo.pt (J.S.); msluna@salud.madrid.org (M.S.-L.); dalia.stoniene@lsmu.lt (D.S.); giulia.vertecchi@uniroma1.it (G.V.); 3 Department of Neonatology and NICU, Ospedale Sant’Eugenio, 00144 Rome, Italy; 4 Division of Neonatology and Pediatrics, Ospedale Versilia, 55043 Viareggio, Italy; luigi.gagliardi@uslnordovest.toscana.it; 5 Maternal and Child Department ASL Bari, Ospedale di Venere, 70131 Bari, Italy; flavia.petrillo@asl.bari.it; 6 Neonatal Intensive Care Unit, Fondazione IRCCS San Gerardo dei Tintori, 20900 Monza, Italy; marialuisa.ventura@irccs-sangerardo.it; 7 Department of Woman’s and Child’s Health, University of Padova, 35122 Padova, Italy; daniele.trevisanuto@unipd.it; 8 Division of Pediatrics, Neonatal Intensive Care Unit and Neonatology, Ospedale dei Bambini “V.Buzzi”, ASST FBF SACCO, 20154 Milan, Italy; 9 TechRes Lab, Department of Electronics, Information and Biomedical Engineering (DEIB), Politecnico di Milano University, 20133 Milan, Italy; raffaele.dellaca@polimi.it; 10 1st Department of Obstetrics and Gynecology, Semmelweis University, 1085 Budapest, Hungary; 11 Department of Pediatrics, Università degli Studi di Siena, 53100 Siena, Italy; 12 Department of Pediatrics, Rea Maternity Hospital, 17564 Athens, Greece; 13 Neonatology Department, University of Medicine Pharmacy Sciences and Technology “George Emil Palade”, 540142 Târgu Mures, Romania; 14 Department of Pediatrics, University of Zagreb School of Medicine, 10000 Zagreb, Croatia; 15 Department of Pediatrics, University Hospital “Majchin Dom”, 1483 Sofia, Bulgaria; 16 Division of Neonatology, Department of Pediatrics, School of Medicine, Gazi University, 06570 Ankara, Turkey; 17 Neonatology Division, Department of Pediatrics, Hospital Beatriz Ângelo, 2674-514 Loures, Portugal; 18 Neonatology Division, Department of Pediatrics, Hospital General Universitario “Gregorio Marañón”, 28007 Madrid, Spain; 19 Department of Pediatrics, Lithuanian University of Health Sciences, LT-50161 Kaunas, Lithuania; 20 Department of Paediatrics, University of Tartu, Tartu University Hospital, 50406 Tartu, Estonia; 21 Department of Pediatrics, Fondazione IRCCS Cà Granda Ospedale Maggiore Policlinico, 20122 Milan, Italy; fabio.mosca@unimi.it; 22 Department of Clinical Sciences and Community Health, University of Milan, 20133 Milan, Italy

**Keywords:** mechanical ventilation, delivery room, preterm infants, RDS, surfactant

## Abstract

(1) Background: Our survey aimed to gather information on respiratory care in Neonatal Intensive Care Units (NICUs) in the European and Mediterranean region. (2) Methods: Cross-sectional electronic survey. An 89-item questionnaire focusing on the current modes, devices, and strategies employed in neonatal units in the domain of respiratory care was sent to directors/heads of 528 NICUs. The adherence to the “European consensus guidelines on the management of respiratory distress syndrome” was assessed for comparison. (3) Results: The response rate was 75% (397/528 units). In most Delivery Rooms (DRs), full resuscitation is given from 22 to 23 weeks gestational age. A T-piece device with facial masks or short binasal prongs are commonly used for respiratory stabilization. Initial FiO_2_ is set as per guidelines. Most units use heated humidified gases to prevent heat loss. SpO_2_ and ECG monitoring are largely performed. Surfactant in the DR is preferentially given through Intubation-Surfactant-Extubation (INSURE) or Less-Invasive-Surfactant-Administration (LISA) techniques. DR caffeine is widespread. In the NICUs, most of the non-invasive modes used are nasal CPAP and nasal intermittent positive-pressure ventilation. Volume-targeted, synchronized intermittent positive-pressure ventilation is the preferred invasive mode to treat acute respiratory distress. Pulmonary recruitment maneuvers are common approaches. During NICU stay, surfactant administration is primarily guided by FiO_2_ and SpO_2_/FiO_2_ ratio, and it is mostly performed through LISA or INSURE. Steroids are used to facilitate extubation and prevent bronchopulmonary dysplasia. (4) Conclusions: Overall, clinical practices are in line with the 2022 European Guidelines, but there are some divergences. These data will allow stakeholders to make comparisons and to identify opportunities for improvement.

## 1. Introduction

Providing appropriate respiratory care for neonates is essential in minimizing complications and remains a critical intervention to decrease the risk of lung and brain injuries, particularly in premature infants, and to reduce mortality. European data from 2014 to 2016 show that around 50% of all babies born between 22 and 32 weeks received a surfactant [1], outlining the need for appropriate skills in the treatment of neonatal respiratory diseases. Bronchopulmonary dysplasia (BPD) affects approximately 40% of extremely preterm infants born at a <29 weeks’ gestational age (GA) [2], and it is associated with short- and long-term adverse consequences. Recent data indicate significant variations in the incidence of BPD among neonatal networks, and these discrepancies may be attributed to differences in care practices [3,4,5].

The present study aimed to survey the practices in respiratory care applied in a large sample of neonatal intensive care units (NICUs) in the European and Mediterranean geographic area. The data obtained in 2022 were evaluated with reference to the recommendations provided in the sixth version of the “European consensus guidelines on the management on the respiratory distress syndrome: 2022 update” [6]. These guidelines are based on the available literature up to the end of 2022 and were developed and endorsed by a panel of experienced European neonatologists and obstetricians.

## 2. Methods

The questionnaire used for the present study is a large cross-sectional electronic survey entitled “European survey on neonatal respiratory care in NICUs” (questionnaire available as Supplementary File S1). The study was submitted to the Ethics Committee of the Azienda Ospedale Università di Padova, which reviewed it and awarded an exemption letter (protocol n. 396n/AO/23), as it did not meet criteria for human-subject research. Research was carried out in line with the principles of the Declaration of Helsinki. The questionnaire was developed by a committee of experts in neonatal pneumology drawn from both the UENPS Scientific Board and the Pulmonology Board of the Italian Society of Neonatology (SIN). A web-based survey was designed following the guidelines set out in the Checklist for Reporting Results of Internet E-Surveys (CHERRIES) [7]. It comprised 89 questions on neonatal respiratory management. The survey addressed five domains: (A) general information; (B) the modes, devices, and strategies employed in the Delivery Room (DR); (C) the modes, devices, and strategies employed in the NICU; (D) the drugs used in the NICU for neonatal respiratory diseases; and (E) mechanical ventilators in the NICU. The items included multiple-choice, fill-in, and “yes/no” questions. For distribution purposes, the presidents or secretaries of all national neonatal societies involved were emailed by the study coordinator on behalf of the President of UENPS, requesting the list of directors of NICUs at the national level. The directors subsequently received an email invitation containing a unique access link to the web-based survey, powered by SurveyMonkey^®^ (San Mateo, CA, USA). Participants were informed of the aims of the survey and that all responses would be anonymized and encrypted before analysis. Completion of the survey encompassed the informed consent for the respondent’s participation. A reminder was sent to non-responders every 3 weeks, for a maximum of 4 times. After that, non-responders were registered as such. In those countries with a restricted policy, where the national neonatal society was not allowed to share contact information (France, Germany, Spain, The Netherlands, Switzerland, Slovakia, Serbia, Croatia, Montenegro, Belgium, Austria, Denmark), the invitation to participate in the survey was distributed at national level through the national society’s mailing list. Directors who accepted to participate gave their consent by writing their email address on an online contact form set up for that purpose. Participation was completely voluntarily. Invitations to the survey were first sent in February 2022 and were closed by July 2022.

## 3. Statistics

For continuous variables, descriptive statistics used median (25–75th centile) for skewed distributions and mean (standard deviation, SD) for approximately normal ones. For discrete variables, frequency distributions and contingency tables were used. Statistical significance tests employed chi-square tests as appropriate, with an alpha level of 0.05, using the statistical package STATA 15.1 (College Station, TX, USA).

## 4. Results

**Figure 1:**
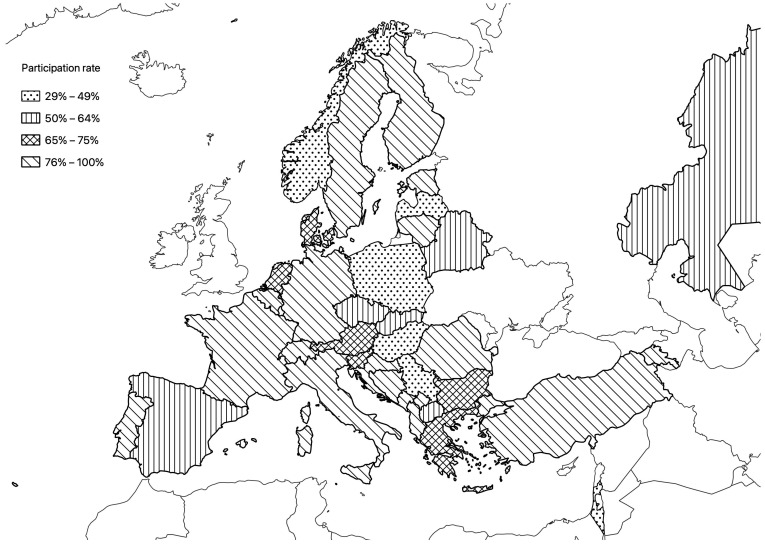
Distribution of the centers.

This survey provides insight into neonatal respiratory care practices in a large sample of NICUs across 37 countries in the European and Mediterranean geographic area (Figure 1). 

The United Kingdom and Ireland were not involved in this study, as their scientific societies did not respond to our invitation. The response rate was 75%: out of 528 units contacted, 410 responded; 13 of these were not NICUs and were excluded from further analysis. At closure, 397 NICUs were included in the study (list available as Supplementary File S2), most of which were also birth centers (93%). Sixty-nine percent of them were academic hospitals. The median (25–75th centile) number of intensive care beds/units of the responding centers was 12 (8–20). Only 32% of the NICUs declared an optimal patient/nurse ratio, i.e., ranging from 1:1 to 2:1. The number of neonates born in the participating centers in 2021 was 1,033,977, with a median of 2300 (1477–3500) per unit. The number of infants admitted to participating NICUs in 2021 was 165,428, with a median of 317 (200–500) per unit. Seventy-six percent of the NICUs admitted over 30 infants with a birth weight < 1500 g, and twenty percent of them admitted over 100.

### 4.1. Devices and Strategies in the Delivery Room

To the question “What is the lowest GA at which you initiate resuscitation?”, 31% of the units replied “22 weeks”, 49% “23 weeks”, and 16% “24 weeks”. Only 15 centres (4%) declared they “initiate resuscitation in infants with GA > 24 weeks”. The device routinely used for respiratory stabilization is the T-piece (68%), followed by variable-flow nasal continuous positive airway pressure (NCPAP) (jet systems) (17%) and the mechanical ventilator (10%). Only 5% of the units are currently using a self- or flow-inflating bag. The facial mask and short binasal prongs (SBP) are the most used interfaces (36% and 35%), followed by the nasal mask (18%). Heated humidified gases for preventing heat loss are used in most of the units (77%).

**Figure 2:**
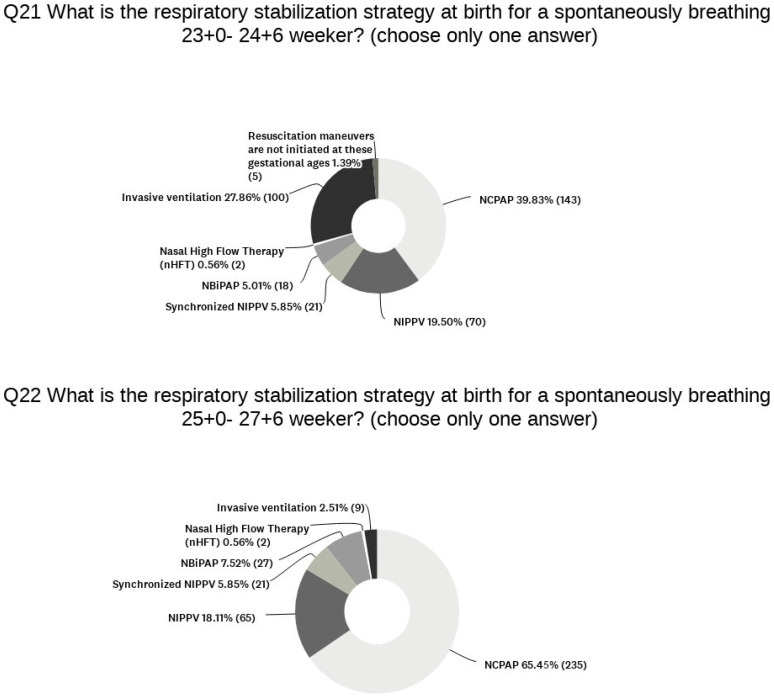
Stabilization strategies for spontaneously breathing 23–24- and 25–26-weeks’ GA premature infants.

Regarding the setting of the initial FiO_2_, almost all the units follow either the suggestions set out by the European Resuscitation Council [8] (49%) or those of the American Academy of Pediatrics (AAP) and American Heart Association [9] (43%), which differ in terms of suggested initial FiO_2_ for infants < 28 weeks GA. Only a few centers use national guidelines (8%). All the units enrolled in the survey aim at a target SpO_2_ of ≥80% within 5 min after birth in newborn infants of <28 weeks GA. SpO_2_ monitoring is performed in 99% of the units and ECG monitoring in 72%; colorimetric capnometry and end-tidal CO_2_ are employed in only 27% of them. A respiratory function monitor (RFM) is used in 21% of the units. The data on the stabilization of spontaneously breathing premature infants with a GA of 23–24 and 25–26 weeks are reported in Figure 2.

**Figure 3:**
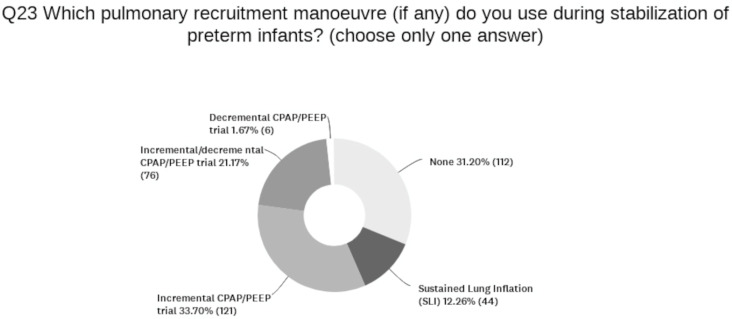
Delivery Room lung recruiting maneuvers.

During stabilization, 69% of the units use a pulmonary recruitment maneuver. DR recruiting maneuvers are reported in Figure 3.

DR surfactant replacement therapy is performed in most of the units (86%), and Less-Invasive-Surfactant-Administration (LISA) and INtubation-SURfactant-Extubation (INSURE) techniques are used with a slightly greater frequency than invasive techniques. Caffeine is frequently (68%) administered in the DR.

### 4.2. Devices and Strategies in the NICU

Regarding non-invasive ventilation (NIV), to the question “What is the main mode used in the NICU?”, most units replied nasal continuous positive-pressure ventilation (NCPAP) (56%), followed by nasal intermittent positive-pressure ventilation (NIPPV) not-synchronized (17%) or synchronized (13%), and bilevel positive airway pressure (BiPAP) (9%). Among the devices for NCPAP, the conventional ventilator is the most frequently used (34%), followed by variable-flow NCPAP (18%) and bubble NCPAP (5%). Synchronized NIPPV (SNIPPV) is most often performed, using a pressure-trigger or flow-trigger. Nasal High-Flow Therapy (nHFT) and nasal high-frequency oscillatory ventilation (NHFOV) are very rarely used (3% and 1%, respectively). SBPs and the nasal mask are the most used interfaces for NIV.

**Figure 4:**
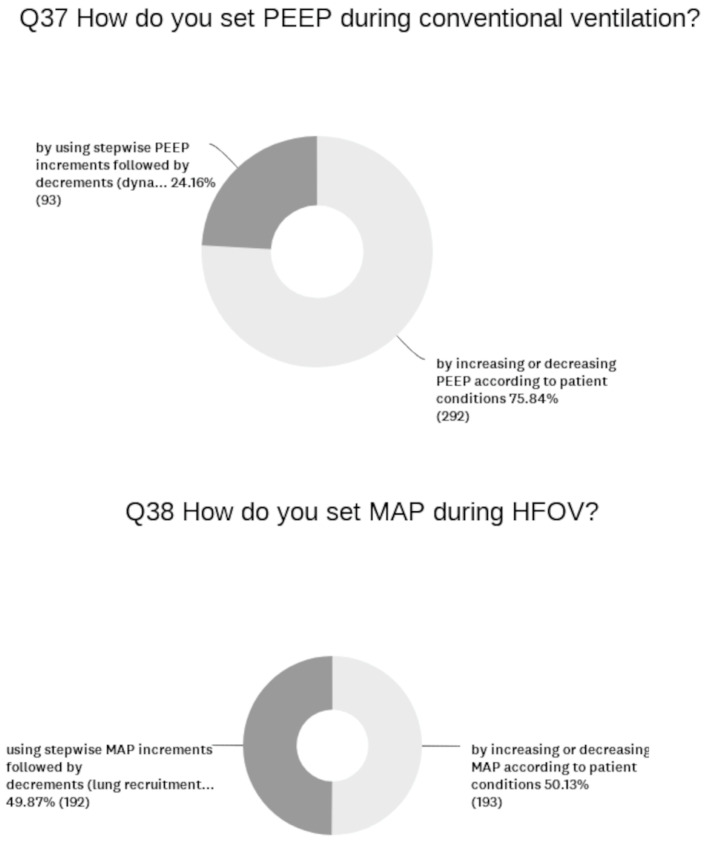
Lung recruitment maneuvers during conventional ventilation and HFOV.

To the question “What invasive ventilation strategy is the first choice for treating acute respiratory distress syndrome (RDS) in preterm infants?”, 63% give volume-targeted synchronized intermittent positive-pressure ventilation (SIPPV + VTV) as their first choice, followed by SIPPV (24%). Tidal volume (Vt) is set at around 5–6 mL/kg by 46% of units, and the rest set Vt according to the disease and postnatal age. By contrast, a high frequency oscillatory ventilation (HFOV) is used in only 11% of the NICUs, of which 5% use a volume-targeted mode (HFOV + VTV). When treating high-risk infants (extremely low gestational age, prolonged-premature rupture of membrane with lung hypoplasia, etc.), 32% of the units change to HFOV or HFOV + VTV as the first treatment choice. The use of lung recruitment maneuvers during conventional ventilation and HFOV is reported in Figure 4.

SIPPV + VTV is also the first choice for weaning; the second and third choices are synchronized intermittent mandatory ventilation + pressure support ventilation (SIMV + PSV) and PSV alone, both associated with VTV.

Closed-loop oxygen control is used in 30% of the NICUs, and 76% of NICUs have a Nitric Oxide dispenser. Less than 35% of the units have written protocols to move from non-invasive to invasive ventilation and vice versa. Only 6% of NICUs have a standardized protocol for performing a tracheostomy in a chronically ventilated preterm infant.

### 4.3. Drugs for Neonatal Respiratory Diseases

#### 4.3.1. Surfactant

**Figure 5:**
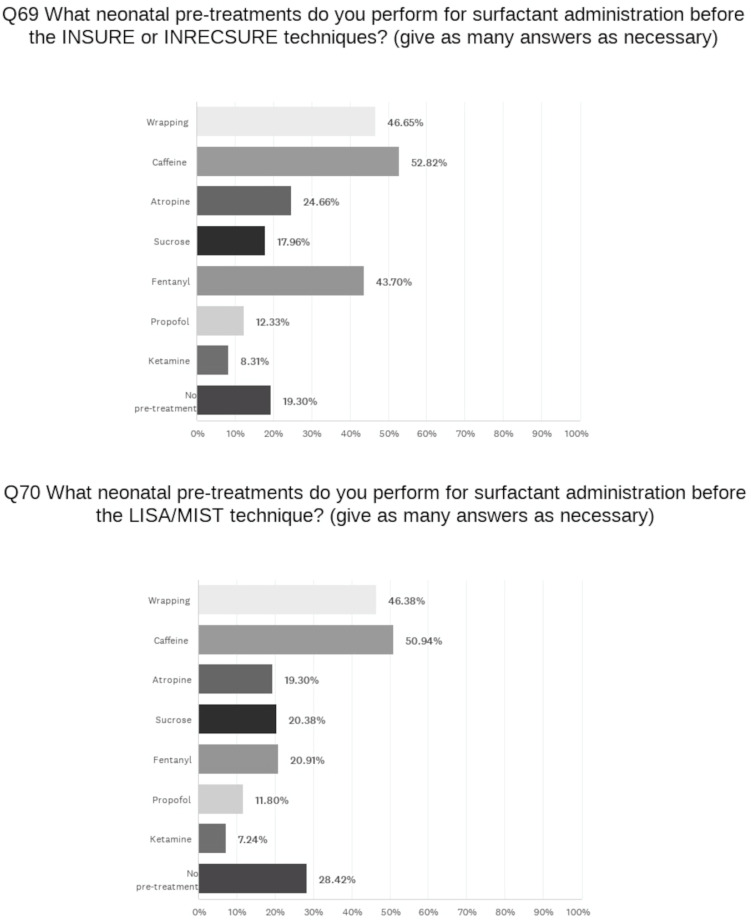
Pre-treatments for INSURE and LISA/MIST approaches.

The aggregate ranking of indicators used by the NICUs to administer surfactant to newborns with RDS on NIV is as follows: level of FiO_2_; SpO_2_/FiO_2_ ratio; chest X-ray; Silverman score; lung ultrasound score. The threshold of FiO_2_ for administration of surfactant is ≤0.3 in 49% of the units (in detail: <0.25 in 5 units (1.3%), 0.25–0.29 in 33 (8.7%), and 0.3 in 150 (39.5%)), 0.31–0.4 in 42%, and ˃0.4 in 9%. About 40% of the units increase the FiO_2_ threshold when treating infants with a higher GA. The natural surfactants used are as follows: porcine (94%); bovine (21%); calf (8%). Synthetic surfactant is used in only 4% of the NICUs. Most of the units (66%) administer the correct dose/kg of the drug regardless of how many vials must be opened. A laryngeal mask (LM) is never used for surfactant administration. An endotracheal tube (ETT) for INSURE and a thin catheter for LISA/MIST (Minimally Invasive Surfactant Therapy) are equally used (48% and 52%) for surfactant administration during NIV. The most used device to perform LISA/MIST is a purpose-built instillation catheter (70%). Most of the units consider infants of ≥24 weeks GA to be suitable for the LISA/MIST technique, with the interval of 26–31 weeks being the most indicated. Only 17% of them use the technique in infants of <24 weeks. In 17% of centers, LISA/MIST is never used. Sixty-two percent of the NICUs perform a lung recruitment maneuver before administering surfactant, increasing CPAP or PEEP during conventional NIV or invasive ventilation, and MAP when using HFOV. The INRECSURE (Intubate-Recruit-Surfactant-Extubate) technique [10] is used in 18% of centers, mostly in Italy where the technique was developed. When administering surfactant through an ETT, 51% of units use a closed circuit. When performing the INSURE technique, 56% of the NICUs remove the ETT immediately after the infant resumes breathing spontaneously, but in 20% of the units, this time varies from 30 min to 2 h or is undefined (24%). The most frequent pre-treatments for INSURE and LISA/MIST are indicated in Figure 5.

Our results show that, in addition to RDS, a surfactant is used for the treatment of many different respiratory diseases of the newborn, such as meconium aspiration syndrome, pneumonia, pulmonary hemorrhage, neonatal ARDS (i.e., bronchiolitis), and also congenital diaphragmatic hernia.

#### 4.3.2. Caffeine

To the question “Which analeptic do you use?”, all the NICUs replied caffeine, and 73% of them use a brand caffeine. The median highest used loading dose reported by the NICUs is 20 mg/kg, and the median highest used maintenance dose is 10 mg/kg. In infants with a birth weight < 1250 g, prophylactic caffeine is started within 2 h of life in 64% of the units. Doxapram is used in 12% of NICUs. Methylxanthines are used in 88% of the NICUs in intubated patients.

#### 4.3.3. Steroids

To the question “In what circumstances do you use steroids in the first 3 weeks of life?”, 72% of the NICUs replied that they use them to facilitate extubation, and 49% of the units use them to prevent the high risk of BPD, regardless of ventilation mode. Most of the NICUs consider administrating steroids in the second (30%) or third week of life (41%), and very few in the first week (3%). Reasons to administer steroids are not only to treat babies who remain on MV in order to facilitate extubation (72%), but also to treat infants at high risk of BPD regardless of the mode of respiratory support (50%). About steroids, low-dose dexamethasone is used in 51% of the NICUs to prevent BPD, while the standard dose is used in 21%. Hydrocortisone is used in 23% of the units. Forty-five percent of the units perform only one cycle of steroids, and 54% of them perform two or even three. Inhaled steroids are employed in 60% of the NICUs.

### 4.4. BPD Incidence

The median (25–75th centile) incidence of severe BPD among <29 weeks GA infants, defined as the need for oxygen and invasive or non-invasive mechanical ventilation (MV) at 36 weeks postconceptional age, is 10% (3.5–23). According to our data, no statistical relationship was observed between the incidence of BPD and the following: the first-choice mode of invasive and non-invasive ventilation in the acute phase of RDS; closed-loop oxygen control use; FiO_2_ threshold for surfactant administration; using LISA versus not using LISA; low-dose versus standard-dose steroids; and single-course versus multiple-courses steroids.

### 4.5. Training

Most of the NICUs (63%) have a policy for upgrading training in respiratory care and techniques, and most of the courses attended are national face-to-face (80%) or web courses (48%). International courses are attended less frequently: 28% via web and 11% face-to-face. Almost all the units would be interested in courses on respiratory care, organized in their specific countries.

## 5. Discussion

The survey results indicate that the devices, strategies, and clinical practices for neonatal respiratory care generally align with the recommendations recently provided in the “European Guidelines for the Management of RDS” published in 2023. Nevertheless, there are some divergences mostly in those fields where the available evidence has not yet led to clear indications and research is ongoing.

Starting from DR resuscitation, there is an increasing inclination to provide initial life support to infants born at lower perceived levels of viability, which varies between 22 and 24 weeks of GA across most of the surveyed countries. Only a minority of the units take care of infants from 25 weeks on. The reasons for these differences can be found in organizational aspects for some of the NICUs, and in cultural, religious, demographic, and economic factors for some others. In the DR, ventilation is most commonly administered through a T-piece with heated and humidified gases. The utilization of the T-piece is linked to higher survival rates without significant morbidities when compared to the self-inflating bag [11,12]. This device enables the delivery of constant PIP and PEEP but not a constant Vt, which is influenced by the lung compliance and inspiratory effort of the infant. Moreover, during mask ventilation, the effective Vt may be conditioned by leaks or airway obstructions [13]. So, because the delivered Vt is highly variable, reliance on pressure alone may be insufficient to provide safe and effective ventilation. The use of an RFM during neonatal resuscitation makes it easier to deliver an appropriate Vt. RFM helps to improve manual ventilation and, thus, lowers the incidence of intraventricular hemorrhage [14,15], and it is useful for teaching and training [16]. However, this device requires experience to be correctly managed. Indeed, caregivers have to quickly interpret and react to different physiological signals during a complex and stressful procedure like DR resuscitation [14].

The existing literature indicates that spontaneously breathing preterm infants should undergo stabilization with CPAP [6,13]. Consequently, this approach is applied consistently, even for infants born at 23–24 weeks of gestational age. The majority of units strive to attain a target SpO_2_ level of 80–85% within 5 min of birth, aiming to enhance both survival rates and overall outcomes [17]. Routine maneuvers for lung recruitment based on CPAP/PEEP trials are widely performed. This represents an important, topical investigational area, and studies are currently underway aiming at defining the best way to improve respiratory transition at birth [18]. A few units are still using sustained lung inflation, which should not be performed as there is no evidence of benefit [19,20]. DR intubation should be reserved to infants not responding to positive pressure ventilation via a face mask or SBP, and the correct placement of the ETT should be verified quickly using a CO_2_ detection device [6,8,9]. Unfortunately, this tool is available only in one third of the units, and its use should be increased. In the majority of DRs, surfactant is administered, with a growing preference for minimally invasive techniques that underscore the idea of gently aiding the transition. Furthermore, the widespread adoption of caffeine prophylaxis has been observed, as earlier treatment is linked to improved outcomes [21]. Compared with a previous survey [22], caffeine use in the DR has increased. A recent feasibility study [23] showed that it is possible to administer enteral caffeine in the DR without interfering with infants’ postnatal assistance, opening the way for further research into its very-early administration.

Our survey shows that the most used non-invasive mode is NCPAP, followed by NIPPV, both of which are recommended by the guidelines in order to minimize MV [6]. The guidelines also suggest that the choice of system for delivering CPAP is of little importance and that BIPAP devices confer no advantage over CPAP [6]. Recent evidence indicates that the synchronization of non-invasive ventilation is a central element in modern ventilatory support and may reduce BPD, supporting infants’ spontaneous respiration more physiologically and optimizing their comfort [6,24].

Data in the literature show that around half of newborns of <28 weeks GA require invasive MV [25]. In addition, around half of these fail their first extubation attempt, thus worsening outcomes [26]. Regarding invasive ventilation strategies, lung-protective modes such as conventional ventilation + VTV or HFOV should be the first choice for babies with RDS who require MV to avoid over-distension and atelectasis [6]. Indeed, most of the NICUs follow these recommendations, using SIPPV + VTV as a first choice to treat RDS. Conventional VTV leads to reduced ventilation, fewer air leaks, and a lower incidence of BPD due to the capability of real-time pressure weaning as lung compliance improves [27]. As reported, when treating high risk infants, one third of the units shift to HFOV and HFOV + VTV as first choices. During weaning, volume-targeted techniques in which all the spontaneous breaths are supported (SIPPV + VTV, SIMV + PSV, PSV + VTV) are correctly [28] and largely employed. It is disappointing to observe that, despite following the best evidence on lung protective ventilation, BPD incidence is still so highly varied between the surveyed units. BPD is acknowledged as an abnormal reparative reaction to both prenatal and postnatal damage affecting the developing lungs [29]. One possible reason for this variability may lie in how closely the various practitioners in a unit follow the guidelines and how well the medical directors of each unit summarized this when responding to the survey on behalf of their fellow clinicians. Another reason is the lack of written policies on ventilation strategies in more than 60% of the NICUs.

Closed-loop automated oxygen control has not gained widespread adoption among units. Despite the fact that this tool has been shown to improve target saturation achievement in short-term trials, whether long-term outcomes will be improved with its use requires further investigation [30].

Evidence shows that tracheostomy in infants with severe BPD who require prolonged high-level respiratory support may improve short- and long-term respiratory and neurodevelopmental outcomes and facilitates timely transition to the home [31]. The advantages of tracheostomy are a lower need for sedation, the ability to move patients out of the PICU, the enhanced parent–child interaction, and improved patient comfort. When clinicians and parents are evaluating the decision of whether to undergo tracheostomy, it is important to consider these potential positive effects. A suggested timing for tracheostomy is before 120 days of life [32].

Among responding units, INSURE or LISA/MIST techniques to administer exogenous surfactant are used roughly equivalently. European guidelines suggest LISA/MIST as the preferred surfactant administration mode, as it improves outcomes by further reducing the need for invasive ventilation compared to INSURE [6]. However, only a few centers use this technique in infants < 24 weeks GA [33]. Similarly, according to our results, the advantages of the technique are more perceived with increasing the infant’s GA. Lack of confidence is the main factor that influences the choice not to perform LISA, and most of the NICUs declare that dedicated training would stimulate its use. Finally, surfactant administration through an LM for infants weighing > 1000 g is a strategy that needs to be implemented [34].

Lung recruitment before surfactant administration improves gas exchange and lung function in animal models of lung injury, owing to a more homogeneous surfactant distribution within the lungs [35]. The importance of lung recruitment has also been shown in human studies [10]. Accordingly, a lung recruitment maneuver by increasing CPAP/PEEP or MAP is frequently carried out before surfactant administration. For infants who receive a surfactant through an ETT, this is given in only half of the NICUs via a closed circuit, and spreading is supported by manual ventilation in a quarter of them. These strategies may be considered as sub-optimal since they may cause lung de-recruitment or overexpansion. Studies are ongoing in these fields [36,37].

Although sedative premedication for endotracheal intubation is considered a standard of care, before LISA/MIST procedure, about 30% of the surveyed units use no pre-treatment. Indeed, for these less invasive surfactant administration techniques, it is not yet clear what is the most appropriate pre-treatment, and the issues related to sedation and the use of the laryngoscope are still open due to the risk of the inhibition of spontaneous ventilation [33]. Early caffeine is a consolidated strategy to treat apnea in the NICUs we surveyed. For infants who do not respond to caffeine, doxapram may confer some benefits [38]. Nevertheless, due to the risk of dose-related adverse events [38], only a few responding units use this medication. Finally, we observed wide variations in administration strategies for postnatal steroids, reflecting the lack of clear guidance in this field. Indeed, the most recent statement of the AAP suggests that neonatologists must continue to use their clinical judgment, balancing the potential adverse effects of corticosteroid with those of chronic lung disease [39].

About training, most units consider it useful to stay up-to-date with tailored courses on respiratory care and be part of a network that allows sharing of knowledge.

### Strengths and Limitations

Our study shows both strengths and limitations. Among its strengths are the structured questionnaire developed by a team of experts in the field. Additionally, the study assessed various aspects of neonatal respiratory management and benefits from a widely distributed representative sample. Its limitations include low response rates from certain areas, potentially introducing selection bias, and information being provided primarily by neonatal ward directors that, coupled with a lack of written protocols or procedure regarding the areas that are queried, may possibly reflect a biased perspective. Nevertheless, in sending our survey to the heads of units, we were confident that the governance of medical directors has a significant influence on the policies followed by their units. Another limitation is that in general, the participating NICUs had a relatively small size, with a low median number of beds and with a volume of neonatal-care <50 VLBW admitted infants per year in half of the centres. It is well known that small numbers of patients do not support the acquisition and retention of expertise [40], and this may be an important contributor to variability in adherence to the guidelines as well as variation in outcomes like BPD. However, this reflects the organization of the perinatal care in the surveyed countries. Despite these limitations, the questionnaire was designed to gather objective and detailed information based on international recommendations, providing insights into hospital policies.

## 6. Conclusions

The results of this survey give information on neonatal respiratory care practices in a large sample of NICUs across 37 countries in the European and Mediterranean area. Overall, clinical practices are in line with the 2022 European Guidelines and, probably, the least-carefully observed are not the most evidence-based. The next step will be to provide the participating National Societies with precise information on RDS management in their own country, offering a means to construct a precise single-nation picture of neonatal RDS care. These data will allow stakeholders to compare national data with the overall trends, to understand differences in clinical practice, to carry out comparative evaluation of outcomes, to identify opportunities for improvement, and to develop a European collaborative platform for staying up to date. Further studies are needed in some areas of assistance like respiratory care strategies and monitoring at birth, lung recruitment maneuvers, non-invasive surfactant administration techniques, and simple, reliable methods of synchronization for NIV, as they may have a significant impact on care.

## Data Availability

The data that support the findings of this study are available from the corresponding author upon reasonable request. Data available on request due to privacy and ethical.

## Supplementary Materials

The following supporting information can be downloaded at: https://www.mdpi.com/article/10.3390/children11020158/s1, File S1: Complete questionnaire; File S2: List of participating centres.
