# Supplementary material for: A Survey of the Union of European Neonatal and Perinatal Societies on Neonatal Respiratory Care in Neonatal Intensive Care Units
Source: Children (Basel). 2024 Jan 26;11(2):158. doi: 10.3390/children11020158 (PMC10887601; doi:10.3390/children11020158)
Supplement: Supplementary file 1 [file children-11-00158-s001.zip › Supplementary files 1 and 2/Supplementary file 1 - Questionnaire.pdf]

European Survey on Neonatal Respiratory Care in NICUs

Presentation

The aim of this survey is to provide a comprehensive picture of the devices and strategies for the care of neonatal respiratory disease in European NICUs. The questionnaire is divided into five sections.

The 5 sections are as follows:

A) General information
B) The modes, devices and strategies employed in your Delivery Room (DR)
C) The modes, devices and strategies employed in your Neonatal Intensive Care Unit (NICU)
D) The drugs used in your NICU for neonatal respiratory diseases
- Surfactant
- Other drugs

E) The mechanical ventilators in your NICU

Survey advisory board:
Camilla Gizzi, Flavia Petrillo, Maria Luisa Ventura, Raffaele Dellaca', Giulia Vertecchi, Luigi Gagliardi, Daniele Trevisanuto, Gianluca Lista, Fabio Mosca, Corrado Moretti

Data Protection Policy:
The data collected by this survey will be used solely by UENPS and only for the purposes of this survey. It will be used to draw up results, and then it will be anonymised before publication.
No data will be shared with third parties.
GDPR policy will apply in full to the survey (https://eur-lex.europa.eu/eli/reg/2016/679/oj).

Estimated time for completion: 30 minutes

Requested data to take the survey:
- Number of admitted infants with BW <1500 grams in 2021
- Total number of admitted neonates in the NICU (best estimate) in 2021
- Number of births in your center in 2021

Useful technical tips:
1) you can change your answers on any survey page until you click the DONE button to complete the survey.
2) you don't need to complete survey in one session: you can answer some questions and return later to finish the rest.
3) if you need help or have any questions, please write to: uenps.survey@gmail.com

European Survey on Neonatal Respiratory Care in NICUs

A. General Information

* 1. Contact Information

Departement/Unit

Hospital

Contact Person Name

City/Town

State/Province

Country

Email Address

Phone

* 2. Academic Hospital

- Yes
- No

* 3. Is your unit a Neonatal Intensive Care Unit (NICU)?

- Yes
- No

European Survey on Neonatal Respiratory Care in NICUs

* 4. Number of intensive care beds in your Unit

* 5. Patient/nurse ratio in your NICU

- 1:1
- 2:1
- 3:1
- 4:1
- 5:1
- Other (please specify)

European Survey on Neonatal Respiratory Care in NICUs

Activity in 2021

* 6. Total number of admitted neonates in the NICU (best estimate) in 2021

* 7. Number of admitted infants with BW <1500 grams in 2021

- Less than 30
- Between 30 and 50
- Between 50 and 100
- Greater than 100

European Survey on Neonatal Respiratory Care in NICUs

* 8. Does your NICU team have a policy for upgrading training on respiratory care and techniques?

◯ Yes

◯ No

European Survey on Neonatal Respiratory Care in NICUs

* 9. If yes, what kind of course your team attend?

- [ ] National web course
- [ ] National face-to-face course
- [ ] International web course
- [ ] International face-to-face course

European Survey on Neonatal Respiratory Care in NICUs

* 10. UENPS wants to organize courses on respiratory care in specific countries. Would a course like this be useful for your NICU?

- ◯ Yes
- ◯ No, there are already a lot of courses in my country

* 11. Is your hospital a birth centre?

- ◯ Yes
- ◯ No

European Survey on Neonatal Respiratory Care in NICUs

* 12. Number of births in your centre in 2021

European Survey on Neonatal Respiratory Care in NICUs

B. The modes, strategies and devices employed in your Delivery Room (DR)

* 13. What is the lowest GA at which you initiate resuscitation? (choose only one answer)

- ◯ 22 weeks
- ◯ 23 weeks
- ◯ 24 weeks
- ◯ 25 weeks
- ◯ 26 weeks
- ◯ Other (please specify)

* 14. What devices are available for respiratory stabilization in non-invasive mode?

- ☐ Self-inflating bag
- ☐ Flow-inflating bag
- ☐ Standard T-piece system
- ☐ Standard jet system (Infant Flow or similar)
- ☐ New jet resuscitation system (R-PAP)
- ☐ Mechanical ventilator

* 15. Which device is routinely used for respiratory stabilization in non-invasive mode? (choose only one answer)

- ◯ Self-inflating bag
- ◯ Flow-inflating bag
- ◯ Standard T-piece system
- ◯ Standard jet system (Infant Flow or similar)
- ◯ New jet resuscitation system (R-PAP)
- ◯ Mechanical ventilator

* 16. What other devices are available for invasive and non-invasive respiratory support? (give as many answers as necessary)

- ☐ Ventilator on transport incubator
- ☐ Conventional mechanical ventilator
- ☐ Hybrid ventilator (conventional + HFOV)
- ☐ Ventilator for non-invasive ventilation
- ☐ High-Flow Therapy device (nHFT)
- ☐ None of these

* 17. Which interface is routinely used for respiratory stabilization in non-invasive mode? (choose only one answer)

- ◯ Facial mask
- ◯ Nasal mask
- ◯ Short binasal prongs
- ◯ Long nasopharyngeal cannulas
- ◯ Double inspiratory loop cannulas (nasal High Flow Therapy models)
- ◯ Single nasopharyngeal tube (cut ETT)

* 18. Which (if any) international guidelines do you follow when setting initial FiO_2?

- ◯ AAP (American Academy of Pediatrics) - AHA (American Heart Association)
- ◯ ERC (European Resuscitation Council)
- ◯ Other (please specify)

[ ]

* 19. Which is your target SpO_2 at 5 minutes in infants with GA <28 weeks? (choose only one answer)

- ◯ 80%-85%
- ◯ 86%-90%
- ◯ >90%
- ◯ I don't have any target

* 20. Do you heat and humidify the gases used for PPV/respiratory support?

- ◯ Yes
- ◯ No

* 21. What is the respiratory stabilization strategy at birth for a spontaneously breathing **<u>23</u>$^{+0}$- <u>24</u>$^{+6}$** weeker? (choose only one answer)

- ◯ NCPAP
- ◯ NIPPV
- ◯ Synchronized NIPPV
- ◯ NBiPAP
- ◯ Nasal High Flow Therapy (nHFT)
- ◯ Invasive ventilation
- ◯ Resuscitation maneuvers are not initiated at these gestational ages

* 22. What is the respiratory stabilization strategy at birth for a spontaneously breathing **<u>25</u>$^{+0}$- <u>27</u>$^{+6}$** weeker? (choose only one answer)

- ◯ NCPAP
- ◯ NIPPV
- ◯ Synchronized NIPPV
- ◯ NBiPAP
- ◯ Nasal High Flow Therapy (nHFT)
- ◯ Invasive ventilation

* 23. Which pulmonary recruitment manoeuvre (if any) do you use during stabilization of preterm infants? (choose only one answer)

- ◯ None
- ◯ Sustained Lung Inflation (SLI)
- ◯ Incremental CPAP/PEEP trial
- ◯ Incremental/decremental CPAP/PEEP trial
- ◯ Decremental CPAP/PEEP trial

European Survey on Neonatal Respiratory Care in NICUs

B. The modes, strategies and devices employed in your Delivery Room (DR)

* 24. In that case what is the **initial** and **min** CPAP/PEEP level (cmH_2O)?

Initial CPAP/PEEP	
Min CPAP/PEEP	

European Survey on Neonatal Respiratory Care in NICUs

B. The modes, strategies and devices employed in your Delivery Room (DR)

* 25. In that case what is the **initial** and **max** CPAP/PEEP level (cmH_2O)?

Initial CPAP/PEEP

Max CPAP/PEEP

European Survey on Neonatal Respiratory Care in NICUs

B. The modes, strategies and devices employed in your Delivery Room (DR)

* 26. How do you set pressure and time parameters for SLI during stabilization?

Pressure (cmH2O)	
Time (sec)	

European Survey on Neonatal Respiratory Care in NICUs

B. The modes, strategies and devices employed in your Delivery Room (DR)

* 27. What CPAP/PEEP level do you set during non-invasive ventilation (NIV) for stabilization (cmH_2O)?

European Survey on Neonatal Respiratory Care in NICUs

B. The modes, strategies and devices employed in your Delivery Room (DR)

* 28. What monitoring technologies are available ?
(give as many answers as necessary)

- [ ] Pulse oximeter
- [ ] ECG monitor
- [ ] Colorimetric capnometry
- [ ] End-Tidal CO2
- [ ] Respiratory Function Monitor (pressure, flow and volume signals, etc..)
- [ ] Mechanical ventilator for monitoring pressure, flow and volume signals

* 29. Do you use surfactant replacement therapy?

- ◯ Yes
- ◯ No

European Survey on Neonatal Respiratory Care in NICUs

B. The modes, strategies and devices employed in your Delivery Room (DR)

* 30. If yes, which administration technique do you use? (give as many answers as necessary)

- [ ] Conventional mode (= intubation + MV)
- [ ] INSURE (INtubation-SURfactant-Extubation)
- [ ] INRECSURE (INtubation-RECruitment with HFOV-SURfactant-Extubation)
- [ ] LISA (Less Invasive Surfactant Administration)/MIST (Minimally Invasive Surfactant Therapy)
- [ ] Laryngeal mask
- [ ] Pharyngeal instillation

European Survey on Neonatal Respiratory Care in NICUs

B. The modes, strategies and devices employed in your Delivery Room (DR)

* 31. Do you use caffeine?

- ◯ Yes
- ◯ No
- ◯ Sometimes

European Survey on Neonatal Respiratory Care in NICUs

C. The modes, devices and strategies employed in your Neonatal Intensive Care Unit (NICU)

* 32. What invasive ventilation modes are available? (give as many answers as necessary)

- [ ] IMV (Intermittent Mandatory Ventilation)
- [ ] IPPV (Intermittent Positive Pressure Ventilation)/CMV (Conventional Mechanical Ventilation)
- [ ] SIMV (Synchronized Intermittent Mandatory Ventilation)
- [ ] SIPPV (Synchronized Intermittent Positive Pressure Ventilation) / AC (Assist Control)
- [ ] PSV (Pressure Support Ventilation)
- [ ] PAV (Proportional Assist Ventilation)
- [ ] NAVA (Neurally Adjust Ventilatory Assist)
- [ ] HFOV (High Frequency Oscillatory Ventilation)
- [ ] Volume Targeted Ventilation for conventional modes
- [ ] Volume Targeted Ventilation for HFOV
- [ ] Jet ventilation

* 33. What invasive ventilation strategy is the first choice for treating acute RDS in preterm infants? (choose only one answer)

- ◯ Non-Synchronized conventional ventilation
- ◯ Synchronized conventional ventilation
- ◯ Synchronized conventional ventilation + Volume Target
- ◯ HFOV
- ◯ HFOV + Volume Target
- ◯ PAV
- ◯ NAVA

* 34. Do you change this first choice strategy when treating high-risk infants (i.e. ELGA, PPROM with lung hypoplasia, chorionamnionitis, premature birth without antenatal steroid prophylaxis)?

- ◯ no
- ◯ Yes (please specify)

* 35. How do you set Inspiratory time (Ti) during conventional ventilation? (choose only one answer)

- ◯ We set Ti according to GA
- ◯ Looking at the infant’s flow signal
- ◯ I don't know

* 36. In respiratory diseases of preterm infants, during volume targeted ventilation, is it correct to set Vt at 5-6 ml/kg?

- ◯ Yes
- ◯ It depends on the phase of the disease and on postnatal age

* 37. How do you set PEEP during conventional ventilation?

- ◯ by increasing or decreasing PEEP according to patient conditions
- ◯ by using stepwise PEEP increments followed by decrements (dynamic PEEP)

* 38. How do you set MAP during HFOV?

- ◯ by increasing or decreasing MAP according to patient conditions
- ◯ using stepwise MAP increments followed by decrements (lung recruitment procedure)

* 39. Do you use ‘closed loop’ oxygen control?

- ◯ Yes
- ◯ No

* 40. Is a Nitric Oxide dispenser available?

- ◯ Yes
- ◯ No

European Survey on Neonatal Respiratory Care in NICUs

C. The modes, devices and strategies employed in your Neonatal Intensive Care Unit (NICU)

* 41. What invasive ventilation strategy is the first choice for weaning infants from mechanical ventilation? (choose only one answer)

- ◯ IMV
- ◯ IPPV/CMV
- ◯ SIMV
- ◯ SIPPV/AC
- ◯ SIPPV/AC + Volume target
- ◯ SIMV + PSV
- ◯ SIMV + PSV + Volume target
- ◯ PSV
- ◯ PSV + Volume Target
- ◯ PAV
- ◯ NAVA
- ◯ HFOV
- ◯ HFOV + VG

* 42. What <u>non-invasive ventilation</u> modes are used? (give as many answers as necessary)

- ☐ Conventional ventilator NCPAP
- ☐ Bubble NCPAP
- ☐ Variable-flow (Jet Systems) NCPAP
- ☐ NBiPAP
- ☐ NIPPV/NIMV
- ☐ Synchronized NIPPV/NIMV by pressure-trigger
- ☐ Synchronized NIPPV/NIMV by flow-trigger
- ☐ Synchronized NIPPV/NIMV by abdominal capsule
- ☐ Synchronized NIPPV/NIMV by NAVA
- ☐ nHFT
- ☐ NHFOV

* 43. What is the main non-invasive mode used? (choose only one answer)

- ◯ Conventional ventilator NCPAP
- ◯ Bubble NCPAP
- ◯ Variable-flow (Jet Systems) NCPAP
- ◯ NBiPAP
- ◯ NIPPV/NIMV
- ◯ Synchronized NIPPV/SNIMV by pressure-trigger
- ◯ Synchronized NIPPV/SNIMV by flow-trigger
- ◯ Synchronized NIPPV/SNIMV by abdominal capsule
- ◯ Synchronized NIPPV/SNIMV by NAVA
- ◯ nHFT
- ◯ NHFOV

* 44. What NIV interfaces are available? (give as many answers as necessary)

- ☐ Facial mask
- ☐ Nasal Mask
- ☐ Short binasal prongs
- ☐ Long nasopharyngeal cannulas
- ☐ Double inspiratory loop cannulas (nasal High Flow Therapy models)
- ☐ Single nasopharyngeal tube (cut ETT)

* 45. Does your NICU have a protocol for moving from non-invasive to invasive ventilation?

- ◯ Yes
- ◯ No, the neonatologist in charge decides individual cases depending on the condition of the patient

* 46. Does your NICU have a protocol for moving from invasive to non-invasive ventilation?

- ◯ Yes
- ◯ No, the neonatologist in charge decides individual cases depending on the condition of the patient

* 47. What is the incidence (%) of severe BPD at 36 weeks post-conceptional age in infants with GA <29 weeks? (BPD = need for oxygen and NIV or MV)

[ ]

* 48. How is ECMO provided? (choose only one answer)

- ◯ It is available in your NICU
- ◯ It is available at your hospital, but not in your NICU
- ◯ It is not available at your hospital, but you follow a standardized protocol for modes and site of transfer
- ◯ It is not available at your hospital, and the neonatal team decides how and where to transfer individual cases depending on need

* 49. Does your NICU have a standardized protocol for performing a tracheostomy in a chronically ventilated preterm infant?

- ◯ No
- ◯ Yes (at what post-conceptional GA)

European Survey on Neonatal Respiratory Care in NICUs

D. The drugs used in your NICU for neonatal respiratory diseases

D.1 Surfactant

* 50. Which parameters do you consider first as an indicator to administer surfactant for newborns with RDS on non-invasive ventilation?
Rank the following parameters (1 = most important; 5 = least important):

- Level of FiO2
- Chest X-Ray
- Lung ultrasound score
- Level of SpO2/FiO2
- Silverman score

European Survey on Neonatal Respiratory Care in NICUs

D. The drugs used in your NICU for neonatal respiratory diseases

* 51. What FiO_2 threshold do you use to administer surfactant in preterm infants?

- FiO2 <0.25
- FiO2 = 0.25-0.3
- FiO2 = 0.3
- FiO2 = 0.3-0.35
- FiO2 = 0.35-0.4
- FiO2 = 0.4
- FiO2 = 0.4-0.45
- FiO2 = 0.45-0.5
- FiO2 = 0.5
- FiO2 >0.5

* 52. Does your FiO_2 threshold change with higher GA?

- No
- Yes

European Survey on Neonatal Respiratory Care in NICUs

D. The drugs used in your NICU for neonatal respiratory diseases

* 53. Please specify

At which GA

Which FiO2 threshold

European Survey on Neonatal Respiratory Care in NICUs

D. The drugs used in your NICU for neonatal respiratory diseases

* 54. Which surfactant do you use?

Porcine: please specify initial dose (mg/kg) ________

Bovine: please specify initial dose (mg/kg) ________

Calf: please specify initial dose (mg/kg) ________

Synthetic: please specify initial dose (mg/kg) ________

Other, please specify initial dose (mg/kg) ________

* 55. When administering surfactant, how do you manage the relation between vials and dosage? (choose only one answer)

- ◯ You give the correct dose/kg regardless of how many vials you must open.
- ◯ You adjust the dosage in order either to use up the surfactant in the vial (increasing the dose) or to avoid opening a new vial (decreasing the dose).

* 56. What device do you routinely use to administer surfactant during non-invasive ventilation? (choose only one answer)

- ◯ Endotracheal tube [for INSURE or INRECSURE]
- ◯ Thin catheter [LISA/MIST]
- ◯ Laryngeal mask
- ◯ Pharyngeal instillation

European Survey on Neonatal Respiratory Care in NICUs

D. The drugs used in your NICU for neonatal respiratory diseases

* 57. What device do you use to perform LISA/MIST?

- ◯ umbilical catheter
- ◯ feeding catheter
- ◯ tracheal aspiration catheter
- ◯ vascular catheter
- ◯ purpose-built surfactant instillation catheter

* 58. At what weeks of GA are infants considered suitable for the LISA/MIST technique? (give as many answers as necessary)

- ☐ <24
- ☐ 24-25
- ☐ 26-27
- ☐ 28-31
- ☐ ≥32
- ☐ LISA is never used

* 59. Do you perform a lung recruitment manoeuvre before administering surfactant? (give as many answers as necessary)

- ☐ No, never
- ☐ Yes, increasing the level of CPAP or PEEP during NIV, before INSURE or LISA/MIST technique
- ☐ Yes, optimizing MAP during HFOV, in course of INRECSURE technique
- ☐ Yes, increasing the level of PEEP (CMV) or MAP (HFOV) during invasive ventilation when extubation is not expected

* 60. At what weeks of GA are infants considered suitable for the INRECSURE technique? (give as many answers as necessary)

- [ ] <24
- [ ] 24-25
- [ ] 26-27
- [ ] 28-31
- [ ] ≥32
- [ ] INRECSURE is never used

* 61. When administering surfactant through an endotracheal tube, what kind of circuit do you use? (choose only one answer)

- ◯ Open circuit
- ◯ Closed circuit

* 62. How do you support surfactant spreading immediately after endotracheal tube administration when extubation is expected? (choose only one answer)

- ◯ by manual PPV
- ◯ by T-piece resuscitator
- ◯ by ventilator PPV
- ◯ I do not use PPV if the infant is spontaneously breathing on CPAP with good respiratory drive

* 63. If you use the INSURE technique, how long do you wait before removing the endotracheal tube? (choose only one answer)

- ◯ Immediately after the infant resumes breathing spontaneously
- ◯ Within 30 minutes
- ◯ Within 1 hour
- ◯ Within 2 hours
- ◯ Time is variable

European Survey on Neonatal Respiratory Care in NICUs

D. The drugs used in your NICU for neonatal respiratory diseases

* 64. How many infants received surfactant through the INSURE technique last month?

Number of infants *(if you don't know write "999")*

* 65. How many infants received surfactant through the INRECSURE technique last month?

Number of infants *(if you don't know write "999")*

* 66. How many infants received surfactant through the LISA/MIST technique last month?

Number of infants *(if you don't know write "999")*

* 67. How many infants received surfactant during MV last month?

Number of infants *(if you don't know write "999")*

European Survey on Neonatal Respiratory Care in NICUs

D. The drugs used in your NICU for neonatal respiratory diseases

* 68. Besides RDS, for which neonatal respiratory diseases do you administer surfactant? (give as many answers as necessary)

- [ ] Transient tachypnoea of the newborn
- [ ] Meconium aspiration syndrome
- [ ] Pneumonia
- [ ] Pulmonary haemorrhage
- [ ] Congenital diaphragmatic hernia
- [ ] Neonatal ARDS (i.e. severe bronchiolitis)
- [ ] Other (please specify)

* 69. What neonatal pre-treatments do you perform for surfactant administration before the INSURE or INRECSURE techniques? (give as many answers as necessary)

- [ ] Wrapping
- [ ] Caffeine
- [ ] Atropine
- [ ] Sucrose
- [ ] Fentanyl
- [ ] Propofol
- [ ] Ketamine
- [ ] No pre-treatment

* 70. What neonatal pre-treatments do you perform for surfactant administration before the LISA/MIST technique? (give as many answers as necessary)

- [ ] Wrapping
- [ ] Caffeine
- [ ] Atropine
- [ ] Sucrose
- [ ] Fentanyl
- [ ] Propofol
- [ ] Ketamine
- [ ] No pre-treatment

* 71. Which factors influence the choice not to perform the LISA/MIST technique in your NICU? (give as many answers as necessary)

- [ ] Lack of confidence with the technique
- [ ] Doubts about outcomes
- [ ] The LISA technique is not considered less invasive than the INSURE technique
- [ ] Doubts about sedation
- [ ] Other (please specify)
- [ ] Low GA of infant
- [ ] High GA of infant
- [ ] None of these: we perform the LISA technique

* 72. Which resources would increase your use of the LISA/MIST technique? (give as many answers as necessary)

- [ ] Dedicated training
- [ ] More information on outcomes
- [ ] More scientific publications on the technique
- [ ] Follow-up data on the technique
- [ ] Other (please specify)

European Survey on Neonatal Respiratory Care in NICUs

D. The drugs used in your NICU for neonatal respiratory diseases

D.2 Other Drugs

* 73. Which respiratory analeptics do you use? (give as many answers as necessary)

☐ caffeine

☐ doxapram

☐ other methyilxantines (please specify)

* 74. Which type of caffeine do you give to infants? (choose only one answer)

○ Galenic caffeine

○ Brand caffeine

○ We do not use caffeine

European Survey on Neonatal Respiratory Care in NICUs

D. The drugs used in your NICU for neonatal respiratory diseases

* 75. What is the highest <u>loading</u> dose of caffeine that you use?
(please express value in base caffeine mg/kg)

* 76. What is the highest <u>maintenance</u> dose of caffeine that you use?
(please express value in base caffeine mg/kg)

European Survey on Neonatal Respiratory Care in NICUs

D. The drugs used in your NICU for neonatal respiratory diseases

* 77. How do you adjust the caffeine dosage mg/kg? (give as many answers as necessary)

- [ ] According to clinical response
- [ ] According to post-menstrual age
- [ ] You never adjust the standard caffeine dosage

* 78. When do you start prophylactic caffeine in infants with a BW <1250g? (choose only one answer)

- ◯ in the delivery room
- ◯ within 2h of life
- ◯ within 12h of life
- ◯ within 24h of life
- ◯ within 3 days of life
- ◯ No prophylactic use
- ◯ Other (please specify)

* 79. Do you use caffeine or other methylxantines in intubated patients?

- ◯ Yes
- ◯ No

* 80. From what postnatal week do you consider administering steroids? (choose only one answer)

- ◯ First week
- ◯ Second week
- ◯ Third week
- ◯ We do not use postnatal steroids
- ◯ Other (please specify)

European Survey on Neonatal Respiratory Care in NICUs

D. The drugs used in your NICU for neonatal respiratory diseases

* 81. In what circumstances do you use steroids in the first 3 weeks of life? (give as many answers as necessary)

- [ ] Need to facilitate extubation
- [ ] High risk of BPD, regardless of ventilation mode
- [ ] Other (please specify)

* 82. Which postnatal steroid do you use to prevent BPD (i.e.in the first 3 weeks of life)? (choose only one answer)

- ◯ Standard dose Dexamethasone (long cycle)
- ◯ Low dose Dexamethasone (short cycle)
- ◯ Hydrocortisone
- ◯ Methylprednisolone
- ◯ Budesonide + surfactant

* 83. How many postnatal steroid cycles do you typically perform for the same infant?

- ◯ Max 1
- ◯ 2-3
- ◯ >3

* 84. Do you use inhaled steroids?

- ◯ Yes
- ◯ No

European Survey on Neonatal Respiratory Care in NICUs

E. The mechanical ventilators in your NICU

* 85. What features do your mechanical ventilators for invasive ventilation have? (give as many answers as necessary)

- [ ] Comprehensive neonatal-through-adult ventilator, used in neonatal mode
- [ ] Flow sensor inserted at the Y-piece
- [ ] Pressure line inserted at the Y-piece
- [ ] Algorithm for volume target ventilation (VTV or VG)
- [ ] Volume controlled ventilation
- [ ] Pneumotachograph
- [ ] Hot-wire flowmeter
- [ ] Pressure trigger
- [ ] Electrical diaphragmatic activity trigger
- [ ] Abdominal capsule trigger
- [ ] I don't know

* 86. Which kinds of HFOV do you have? (give as many answers as necessary)

- [ ] with active exhalation generated by a piston
- [ ] with active exhalation generated by a Venturi effect
- [ ] with active exhalation generated by another method
- [ ] I don't know

* 87. How are aerosolized drugs administered during invasive or non-invasive ventilation? (give as many answers as necessary)

- [ ] Using jet nebulizers
- [ ] Using vibrating mesh nebulizers
- [ ] Using ultrasound nebulizers
- [ ] Through the ventilator circuit
- [ ] Outside the ventilator circuit
- [ ] We do not administer inhaled drugs during ventilation

* 88. What gas conditioning mode do you use most?

- ◯ 'Automatic' mode for invasive and non-invasive ventilation
- ◯ ‘Set up’ mode with the temperature set manually

* 89. Do you have a standardized protocol for the maintenance of respiratory devices?

- ◯ Yes
- ◯ No
